# Alzheimer’s Disease Detection Using Comprehensive Analysis of Timed Up and Go Test via Kinect V.2 Camera and Machine Learning

**DOI:** 10.1109/TNSRE.2022.3181252

**Published:** 2022-06-15

**Authors:** Mahmoud Seifallahi, Afsoon Hasani Mehraban, James E. Galvin, Behnaz Ghoraani

**Affiliations:** Department of Computer and Electrical Engineering and Computer Science, Florida Atlantic University, Boca Raton, FL 33431 USA; Department of Occupational Therapy, Faculty of Rehabilitation Sciences, Iran University of Medical Sciences and Health Sciences, Tehran 14496-14535, Iran; Comprehensive Center for Brain Health, Department of Neurology, University of Miami, Miami, FL 33136 USA; Department of Computer and Electrical Engineering and Computer Science, Florida Atlantic University, Boca Raton, FL 33431 USA

**Keywords:** Alzheimer’s disease (AD), timed up and go (TUG), Kinect V.2 camera, skeletal data, machine learning, support vector machine (SVM)

## Abstract

Alzheimer’sdisease (AD) is a progressive neurodegenerative disease affecting cognitive and functional abilities. However, many patients presume lower cognitive or functional abilities because of aging and do not undergo clinical assessments until the symptoms become too advanced. Developing a low-cost and easy-to-use AD detection tool, which can be used in any clinical or non-clinical setting, can enable widespread AD assessments and diagnosis. This paper investigated the feasibility of developing such a tool to detect AD vs. healthy control (HC) from a simple balance and walking assessment called the Timed Up and Go (TUG) test. We collected joint position data of 47 HC and 38 AD subjects as they performed TUG in front of a Kinect V.2 camera. Our signal processing and statistical analyses provided a comprehensive analysis of balance and gait with 12 significant features for discriminating AD from HC after adjusting for age and the Geriatric Depression Scale. Using these features and a support vector machine classifier, our model classified the two groups with an average accuracy of 97.75% and an F-score of 97.67% for five-fold cross-validationand 98.68% and 98.67% for leave-one-subject out cross-validation. These results demonstrate the potential of our approach as a new quantitative complementary tool for detecting AD among older adults. Our work is novel as it presents the first application of Kinect V.2 camera and machine learning to provide a comprehensive and quantitative analysis of the TUG test to detect AD patients from HC. This study supports the feasibility of developing a low-cost and convenient AD assessment tool that can be used during routine checkups or even at home; however, future investigations could confirm its clinical diagnostic value in a larger cohort.

## I. Introduction

**A**LZHEIMER’S disease (AD) is a progressive neurodegenerative disease that leads to progressive cognitive and functional decline and other complications such as an increased risk of falling [1], [2]. The number of people with AD is rapidly increasing with the aging population. It now exceeds 35 million worldwide, highlighting the need for accurate and low-cost diagnostic methods for the disease [3]. Current diagnosis relies on collecting a significant amount of data via patient and caregiver interviews, questionnaires, physical exams, neuropsychological tests, brain imaging (i.e., Magnetic resonance imaging (MRI), and other laboratory tests (i.e., electroencephalography (EEG) [1]. However, most of these methods are performed less frequently in routine clinical practice as they can be time-consuming, expensive, and require trained clinical staff to collect data and provide clinical interpretation. Older adults and their families may ignore early warning signs, instead considering declining cognitive and physical function as the symptoms of aging [1]. Developing low-cost and accurate AD risk assessment methods using technology is essential for fast and widespread detection of the disease in clinical or home settings [4].

Previous studies have shown that there is a relationship between gait and cognition suggesting gait as an emerging diagnostic tool for detecting various types of dementia like AD and Parkinson’s disease dementia [5], [6]. Consequently, various types of gait and balance tests like the Berg Balance Scale (BBS), Timed Up and Go test (TUG), and straight and roundtrip walking have been explored for the assessment of AD or other types of dementia [7]. Among these tests, the time of TUG test has shown to be associated with cognitive decline in people with dementia or even mild cognitive impairments (MCI) [8]. This quick and simple test includes several subtasks: standing up from an armchair, walking forward for 3 meters, turning around, walking back to the chair, and sitting down on the chair [9].

In many cases in AD research, results have focused only on the overall time of the TUG test measured using a stopwatch [7], [9]–[11]. However, the TUG test is composed of several different subtasks of standing up, walking, turning, and sitting, which their comprehensive analysis may significantly increase this simple test’s potential to be used as a complementary tool for increasing the sensitivity of AD detection, particularly in its earliest manifestations [12]. Also, the detailed analysis of TUG can provide comprehensive information about the functional and balance levels of older adults with AD [12]. Nevertheless, a few studies have examined the TUG in detail for AD assessments. Mirelman *et al.* [12] and Wang *et al.* [13] used inertial sensors with accelerometers and gyroscopes mounted on the subjects’ bodies as they were performing the TUG and examined subtasks separately. In another study, Ansai *et al.* [14] used a complicated system with seven cameras and placed markers on the subjects’ bodies as they performed the TUG test. Although these studies suggest differences in TUG subtasks between healthy controls (HC) and cognitively impaired subjects, none explored the potential of using a comprehensive assessment of TUG subtasks for discriminating AD patients from HC. Instead, results were largely based on descriptive statistical analysis to examine significant differences in TUG subtasks between the two groups. Moreover, those studies were based on mounting inertial sensors or some markers on the subjects’ bodies that can be sensitive to the sensor/marker placement or use a complicated recording system, limiting its applicability in non-clinical settings. In this paper, we addressed these limitations by developing a novel technology-based approach for comprehensive analysis of TUG or order to differentiate AD patients from HC. Our contribution is two folds: (1) capturing a comprehensive assessment of different subtasks of TUG using a single Kinect V.2 camera and signal processing; and (2) discriminating AD from HC based on their TUG performance using machine learning. Our novel contribution collected joint position data recorded from 47 HC and 38 subjects with AD using a single Kinect V.2 camera as a depth camera introduced by Microsoft without placing any sensor or marker on the subjects’ bodies. Using this data, a comprehensive collection of features was extracted from different subtasks of TUG via analyzing the changes of the position and angles of the joints. Finally, statistical analysis methods were used to identify the TUG subtask features that were significantly different between the two groups, which were then fed into a machine learning method to discriminate AD from HC. Our developed approach based on an affordable and easy-to-use technology and a simple TUG test may not be a standalone diagnostic tool but has the potential to become a first-stage AD evaluation tool to identify AD individuals at early stages and direct them to comprehensive clinical assessments as needed.

## II. Materials and Methods

This section describes the details of our new data and the signal processing and machine learning methods used to process the data.

### A. Subjects

A total of 42 AD and 50 HC subjects were recruited in this study. The AD subjects were recruited from the patients of Iran Dementia and Alzheimer’s Association (IDAA) who underwent comprehensive diagnostic processes and assessments of psychological tests, MRI, and EEG. The HC subjects were recruited from older adults who underwent various checkups and diagnostic protocols as part of the AD prevention program at IDAA. All subjects completed Persian versions of the Mini-Mental State Examination (MMSE), Montreal Cognitive Assessment (MoCA), and clinical depression was excluded using Geriatric Depression Scale (GDS). Subjects experiencing cognitive or functional disorders because of other diseases (e.g., stroke, knee/hip replacement) were excluded from the present study. The Persian version of GDS was used to measure the depression level of the participants. The range of 0–4 was normal, 5–9 mild depression, 10–11 moderate depression, and 12–15 severe depression. Participants with severe depression were excluded from the present study. This study was approved by the ethics committee of Semnan University of Medical Sciences of Iran, and signed informed consent was obtained before subjects participated in the study.

The data processing step rejected some of the participants’ data due to excessive noise or the presence of outliers. After this process, 47 HC and 38 AD subjects’ data were analyzed. Table I provides the demographic and some clinical information of these study participants. Significantly different characteristics (p-value *<*0.05) are indicated with an asterisk.

### B. Recording Tools and Setting

Data collection was performed using a Kinect V.2 camera mounted on a tripod located in the front of the subjects performing the TUG test. The Kinect V.2 was connected to an ASUS-FX503 laptop with Intel Core i7-7700HQ CPU@2.80 GHz 2.80 GHz processor and 8.00GB of Installed Memory (RAM) was used to record and process data at 20 frames/second. The Kinect V.2 camera can record RGB (Red-Green-Blue color model), depth, and skeletal data of 25 joints of persons’ bodies [15], [16]. In the present study, we used skeletal data because it can provide comprehensive movement information without the need for extensive data analysis or storage space. The skeletal data also reduces the privacy concerns with RGB recordings. MATLAB 2019 was utilized for recording and processing data.

**Fig. 1.**
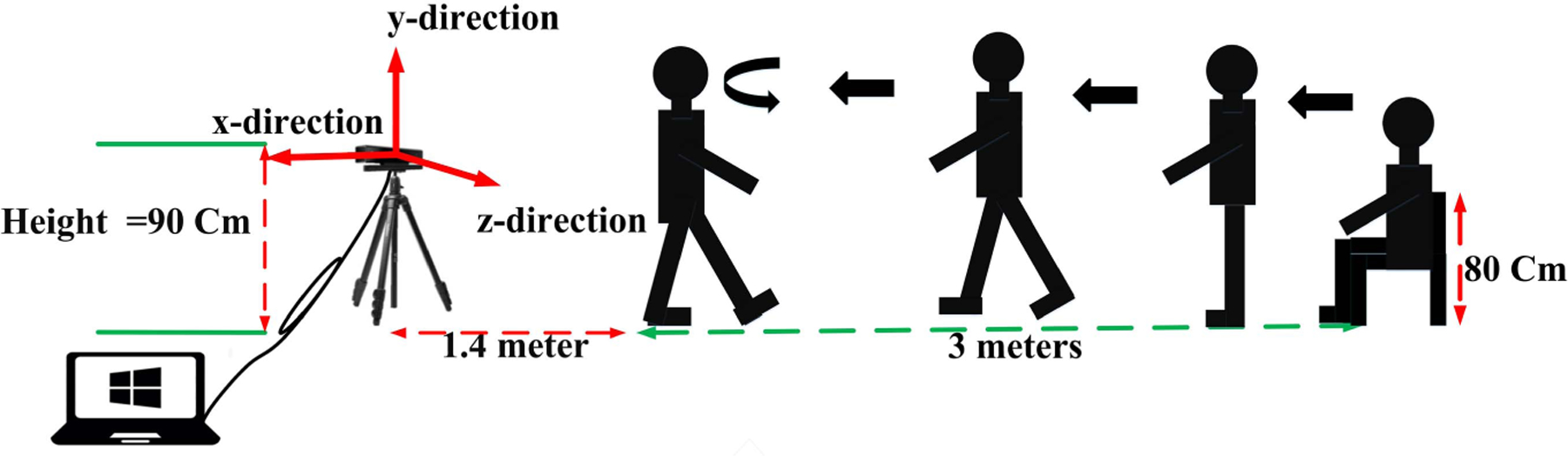
Recording tools and setting for Timed Up and Go test.

**Fig. 2.**
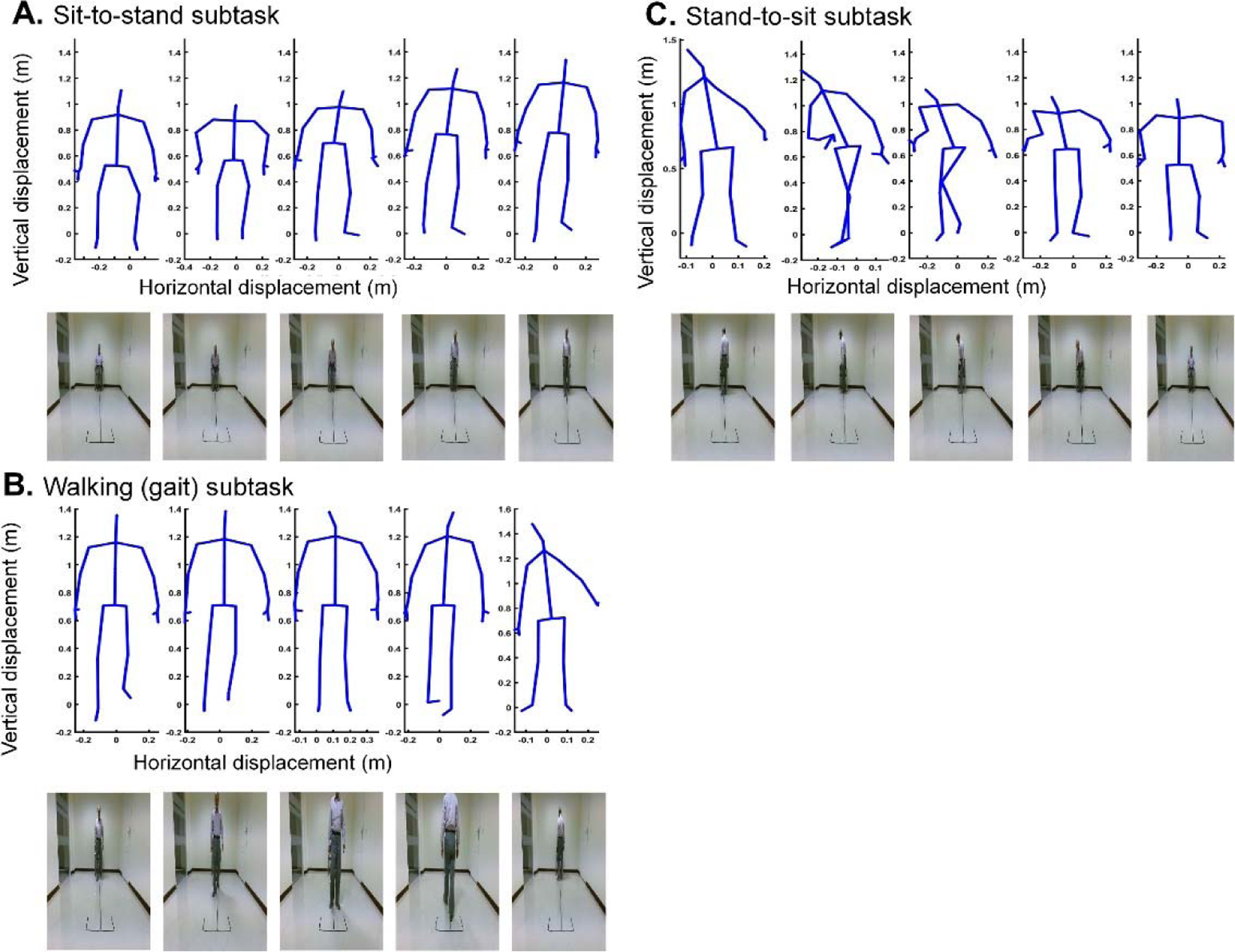
Recorded RGB and skeletal data for the various subtasks of the TUG test using Kinect V.2 camera.

Fig.1 shows the setting of the recording apparatuses and a sample of recorded data. On the day of data recording, the structure of the TUG test was described to the participants. They practiced TUG two to three times before performing the actual test with about three minutes resting between performances. The subjects started their test after being ready and hearing “Go” from the operator who recorded the data [8]. Fig.2 shows skeletal and RGB recorded data for a subject during various subtasks of the TUG test. We placed the Kinect V.2 camera in front of the participants to ensure that all the 25 joints of the body are detected and tracked without any occlusion. Dolatabadi *et al.* [17] have confirmed that the frontal view recordings from a Kinect camera can be used to accurately measure gait features when compared to the gold-standard computerized walkways. Other studies also suggested using the frontal-view recordings for extracting gait features [18]–[22]. When the left or right-side recording is used, some of the joints like shoulder center will be occluded, and as a result, their positions will be estimated from the other joints’ data, which may decrease their accuracy.

Several precautions were made to prevent the distortion of the detected joints. When a person is out of range, the camera estimates the joints’ positions, so we made sure that the person was always in the camera’s full view. Another consideration was to ensure that only the subject performing TUG is in the camera view. Otherwise, the camera reidentifies the new person and this process could affect the quality of the recorded data. We also used curtains to avoid direct sunlight as our investigation showed that excessive sunlight could affect data quality. Furthermore, we repeated the recording process if we noticed any problems with tracking the joints because of the participant’s clothes or any hardware-related issues with the camera or laptop.

### C. Data Processing

**Fig. 3.**
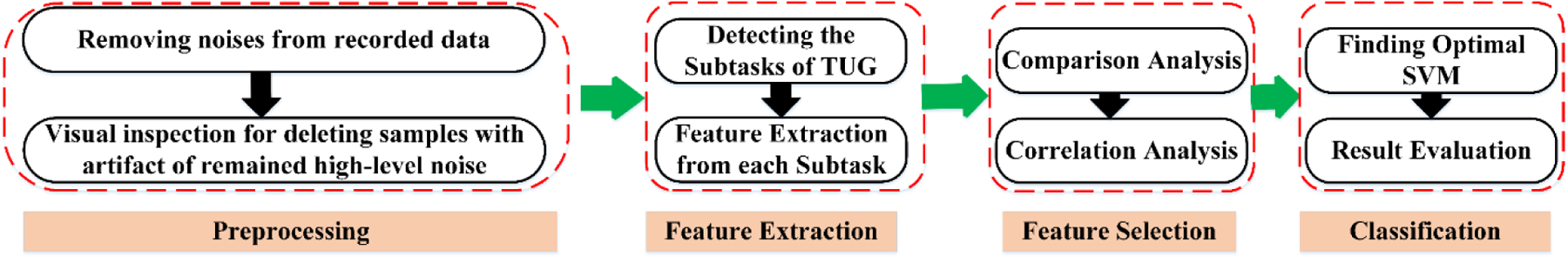
Overall steps of the proposed algorithm for processing and analyzing the recorded skeletal data of TUG test for AD detection.

We performed several successive steps to preprocess the recorded skeletal data, segment the subtasks, and extract a set of comprehensive features from each subtask. Next, the significant features for discriminating HC and AD groups were identified based on the statistical analysis of the extracted features. Finally, a classifier based on a support vector machine (SVM) was developed for the classification of subjects into HC and AD. Fig.3 shows the overall structure of our method, and the details are provided in this section.

#### 1) Preprocessing:

The location of each 25 joints during the recordings was represented as a signal. In the preprocessing step, we removed the noise using a six-order Butterworth filter with a cut-off frequency of 3 Hz as suggested by several studies like Ma *et al.* [23] and Nixon *et al.* [24]. Some samples of the filtered signals are provided in the Supplementary document Section 1. Next, we inspected the signals visually and identified the recordings with a significant amount of noise to the extent that we could not detect the stance or swing phases reliably. For this purpose, we looked through the filtered signals of the right ankle and removed them from the analysis if the filtered signal had abrupt transitions during the stance and swing phase. This process resulted in the removal of the data for 4 AD and 3 HC participants. Examples of these rejected signals are shown in the Supplementary document S2.

#### 2) Segmentation:

The following steps were performed to segment the sit-to-stand, walking, turning, and stand-to-sit subtasks of the TUG test.

**Fig. 4.**
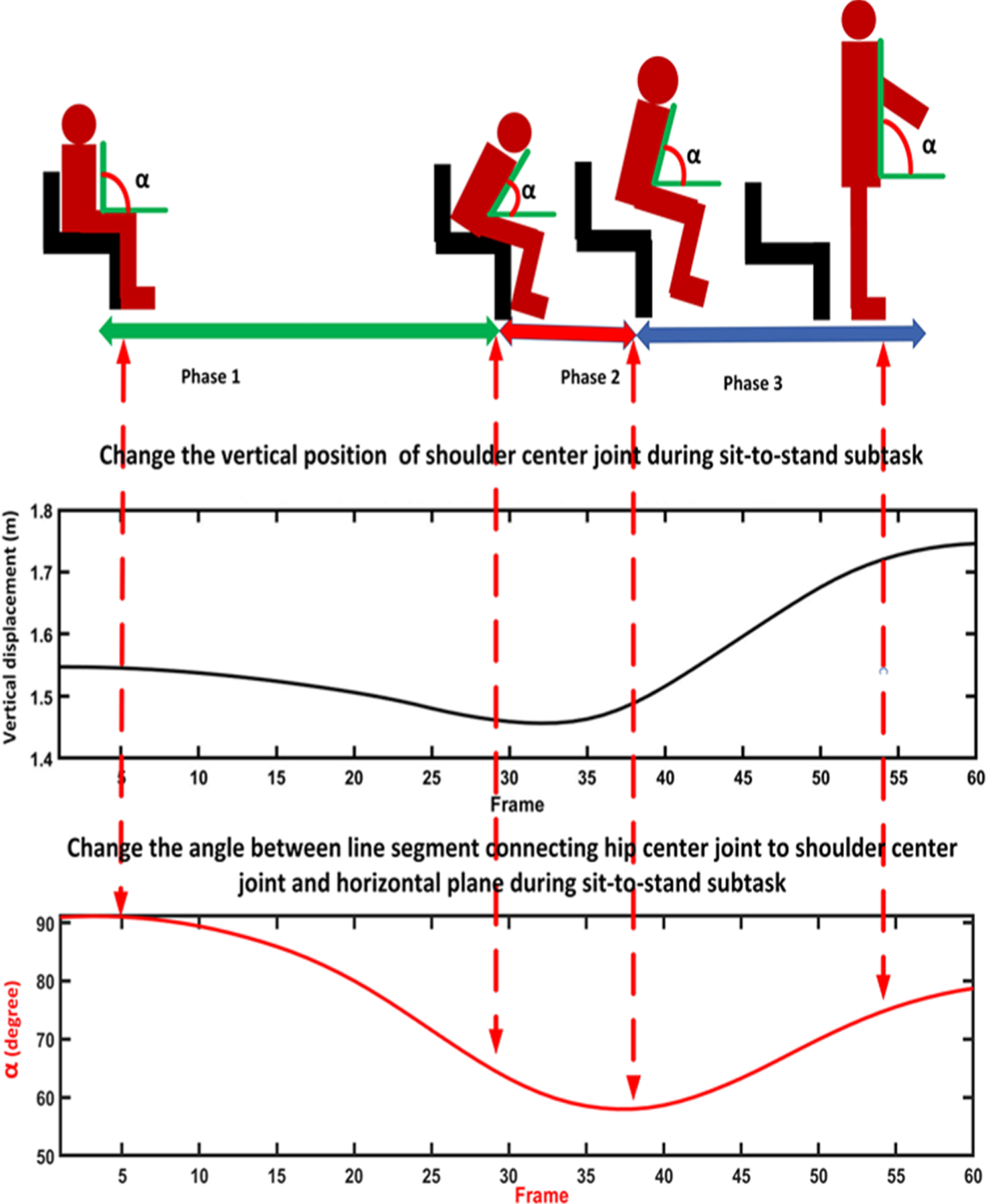
Detection of the sit-to-stand subtask based on the changes of the vertical displacement of the shoulder center joint and the angle α. The angle α is defined between the hip-shoulder center vector and a hypothetical horizontal plane going through the hip center.

**Fig. 5.**
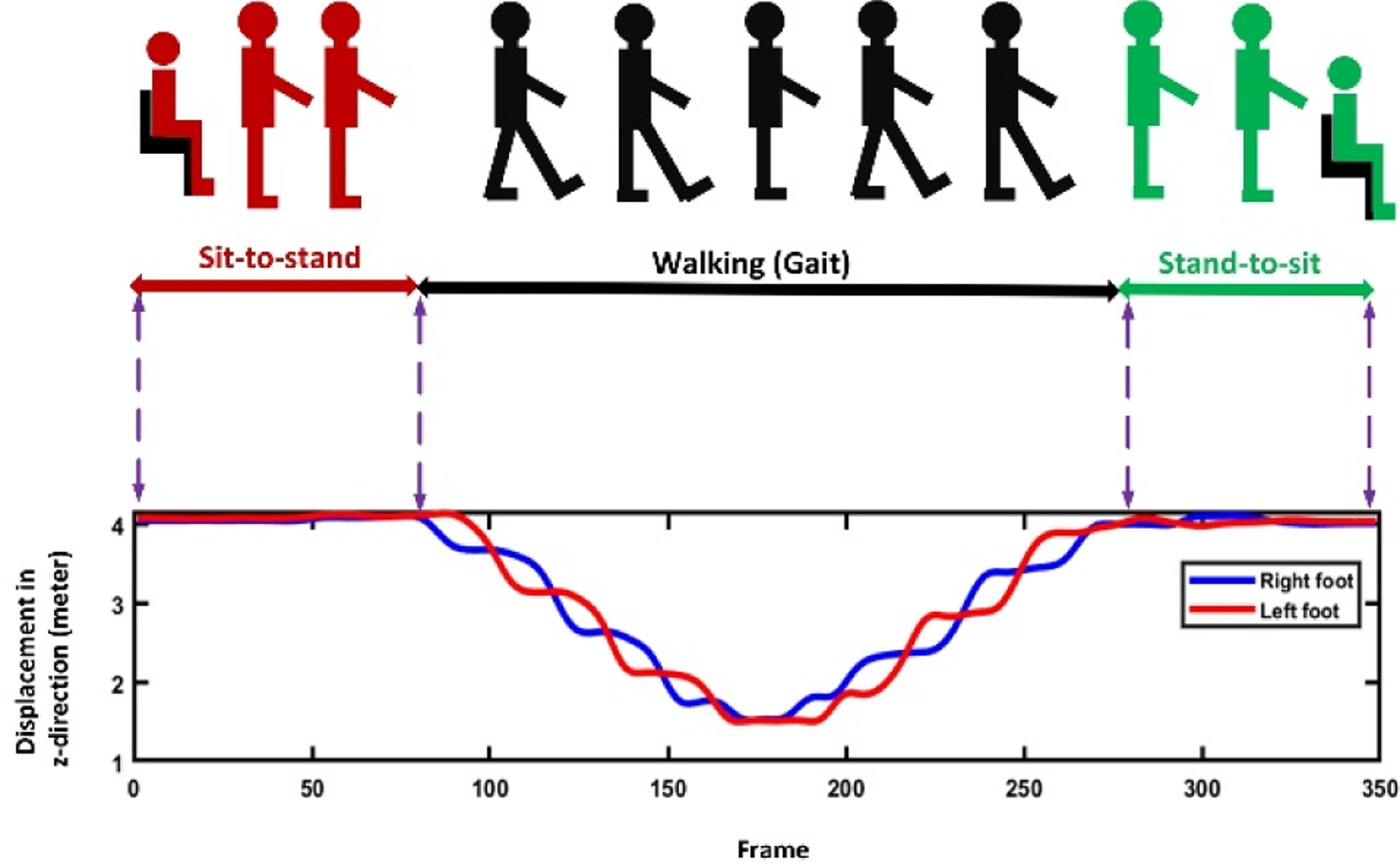
The changes of the position of the right and left feet in the z-direction during different TUG subtasks.

**Fig. 6.**
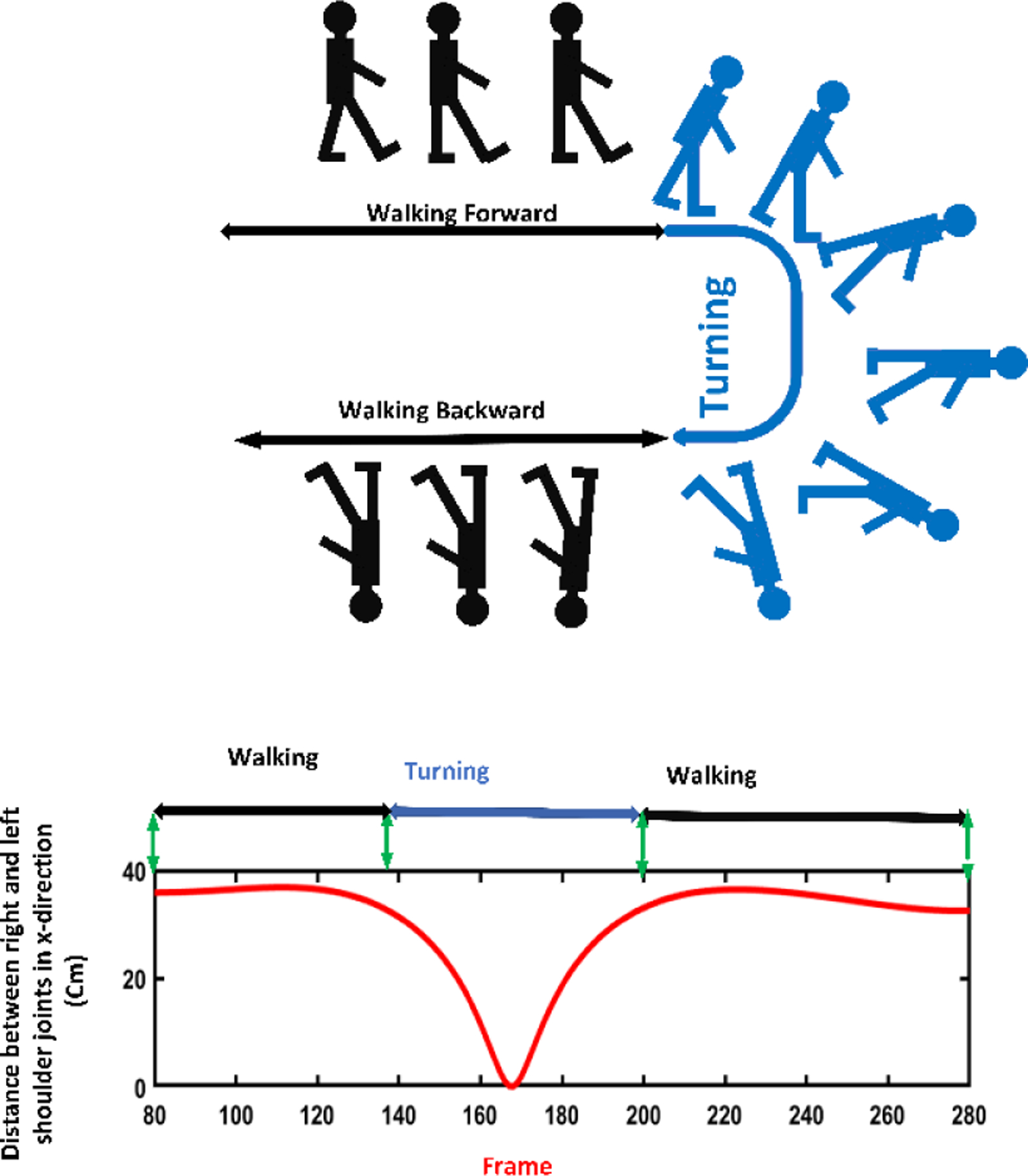
Distance between the right and left shoulder joints in the x-direction during TUG test.

**Fig. 7.**
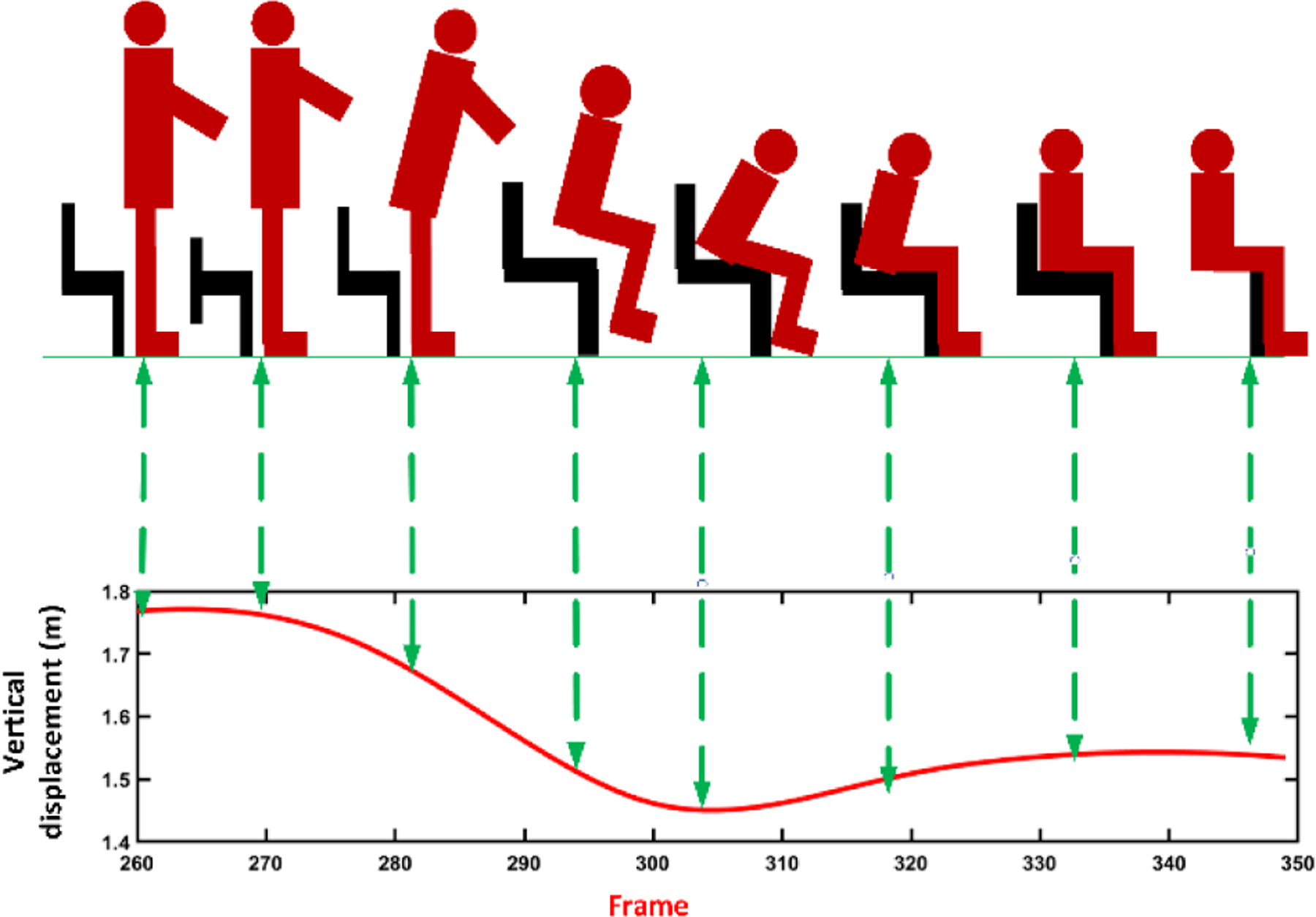
Detection of the stand-to-sit subtask based on the changes in the shoulder center joint in the y-direction.

- **Sit-to-stand subtask:** The change in the position of the shoulder center joint in the vertical direction (y-axis) was used to segment the sit-to-stand subtask. The vertical position of the shoulder center joint remains approximately the same till the person changes their position from sitting to standing. At this point, the vertical position of the shoulder center joint increases to its maximum after a local minimum, representing the bending that happens from sitting to standing. This pattern was confirmed by investigating the changes in the angle between a vector from the hip to the shoulder center joint and a hypothetical horizontal plane through the hip. When changing the position from sitting to standing, thus angle changes from a constant value to a local minimum and then to a constant value [18], [25], [26]. Fig.4 shows changes in the shoulder center joint displacement in the vertical direction (y-axis) and angle during the sit-to-stand subtask.
- **Walking subtask:** After standing up from the chair, the person walks for 3 meters and turns back, and returns to the chair. During this subtask, the vertical position of the shoulder center joint remains approximately constant while the position of the right and left foot joints change in the z-direction [27]. Fig.5 shows the signals of the right and left foot joints in the z-direction while the Kinect V.2 camera is located in front of the test path.
- **Turning subtask:** We used the distance changes between the right and left shoulder joints in x-direction to detect the turning phase from the skeletal data according to the method proposed by Lohmann *et al.* [25]. Fig.6 shows the changes in distance signal of the right and left shoulder joints in the x-direction for detection of turning subtask.
- **Stand-to-sit subtask:** The vertical position change of the shoulder center joint was used to detect the stand-to-sit subtask. The process was similar the sit-to-stand analysis but with reverse actions. The vertical position of the shoulder center decreases gradually from a steady value to a local minimum. It then increases slightly to reach a stable value when a person is in the stable sitting position [25]. Fig.7 shows the changes in the shoulder center joint in the y-direction during the stand-to-sit subtask.

#### 3) Feature Extraction:

A total of 61 features were extracted from the TUG test, out of which 54 were the gait features extracted from the walking subtask and the rest were from the overall TUG, sit-to-stand, turning, and stand-to-sit subtasks. The overall time of the TUG test was calculated using Eq. (1) with a recording rate of 20 frames/second.


(1) $$T_{TUG}=\frac{\text{Total}\;\;\text{number}\;\;\text{of}\;\;\text{recorded}\;\;\text{frames}\;\;\text{during}\;\;\text{TUG}}{\text{Recording}\;\;\text{rate}}$$

The duration and vertical velocity of the sit-to-stand and stand-to-sit subtasks were calculated. Eq. (2) shows the calculation of the vertical velocity as the rate of change in the shoulder center in the y-direction. $Y_{t1}$ and $Y_{t2}$ show the position of the shoulder center joint in the y-direction at the start, $t_{1}$, and end, $t_{2}$, of the subtask respectively.


(2) $$\text{Vertical}\;\;\text{velocity}=\frac{Y_{t2}-Y_{t1}}{t_{2}-t_{1}}$$

**Fig. 8.**
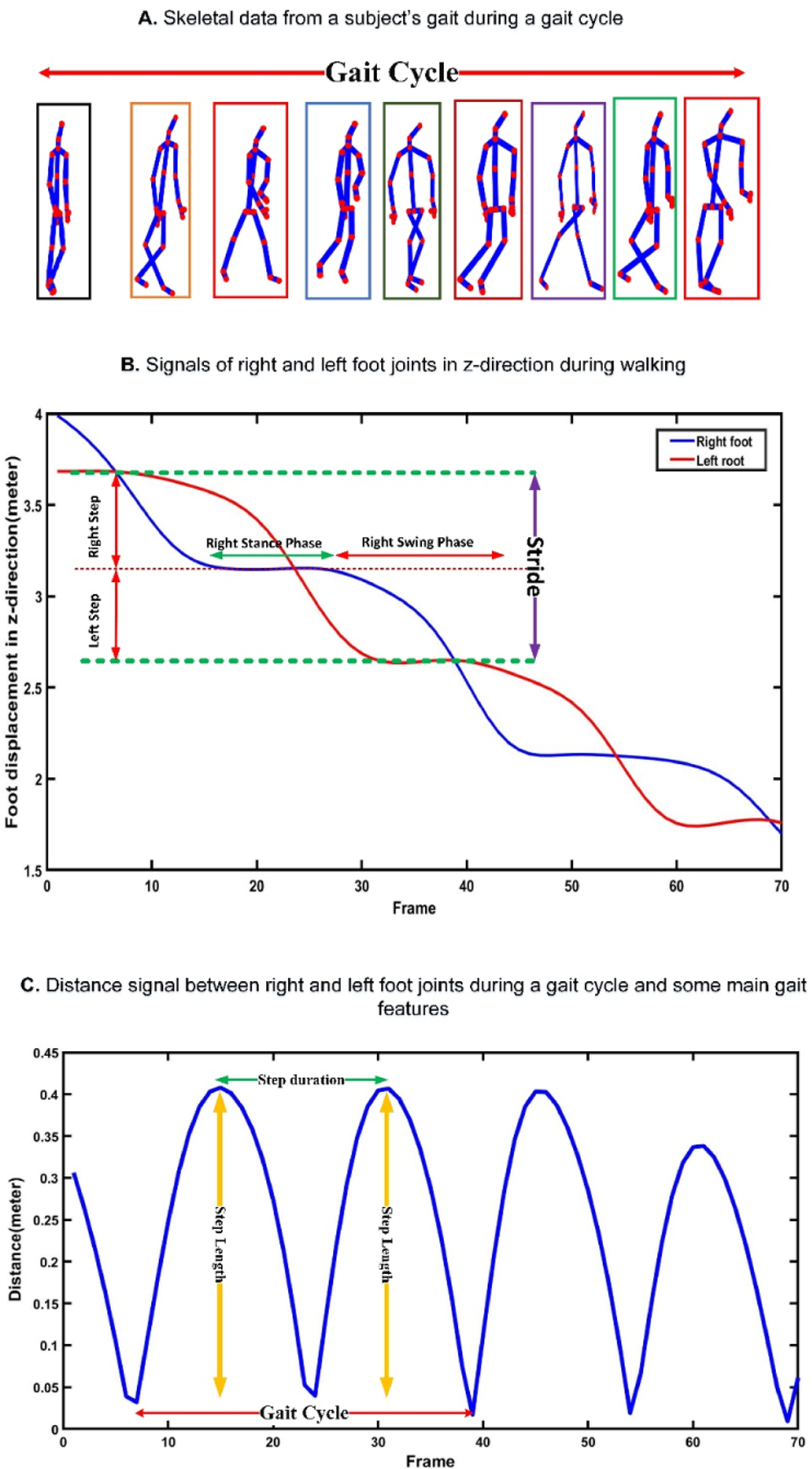
Extracting various walking parameters from the walking subtask of the TUG test based on the changes in the position of the right and left foot joints in the z-direction.

A comprehensive collection of features was extracted from the walking subtask consisting of the duration and average velocity and a wide range of features from step, stride, and gait cycles. To detect steps and strides, the distance signal of the right and left feet was calculated. The local peaks show the steps of subjects’ walking [27]. The right and left feet have two states during a gait cycle named stance and swing phases, which can be automatically detected via analyzing the position of the feet in the z-direction [18]. The gait cycle is defined as the duration where the same foot contacts the ground two times during walking successively. During walking, when a foot is in a stance phase, its location does not change, while its location changes during the swing phase. The stance and swing phases can be calculated using the numerical derivative of the right and left foot signals when a person is walking [18]. Fig.8 shows a part of the right and left foot signals and their distance signal with some important walking features which were extracted from them.

Some statistical values of variability, similarity, and regularity were also calculated for the walking features of the time, length, and velocity of steps and strides. Eq. (3) shows the variability of a feature as the ratio of its standard deviation to mean value [28].


(3) $$\text{CoV}\left(x\right)=\frac{\text{STD}\left(x\right)}{\text{Mean}\left(x\right)}*100$$

Eq. (4) calculates the similarity of steps (i.e., regularity of strides) which measures the similarity between successive steps (strides) during walking [29], [30]. In this equation, $x_{Right}$ and $x_{Left}$ represent the right and left foot feature, respectively.


(4) $$\text{SI}\left(x\right)=1-\frac{\left|x_{Right}-x_{Left}\right|}{\max\left(x_{Right},x_{Left}\right)}$$

Overall, 61 features were extracted from the recorded TUG test for each subject. In addition to the overall time of TUG, two features from sit-to-stand, 54 features from walking (gait), two features from turning, and two features from stand-to-sit subtasks were extracted.

#### 4) Adjusting Features:

The statistical analysis confirmed that age and GDS score are significantly different between the AD and HC cohort. Hence, we used analysis of covariance (ANCOVA) to adjust the extracted features for these two confounding factors [2]. The feature selection analysis in the next subsection was performed on the initially extracted TUG features and the adjusted ones.

#### 5) Feature Selection:

The feature selection was performed in two ways. First, we determined the significant unadjusted and adjusted features based on the entire dataset. This was done to understand the relationship of each feature with the cognitive impairment in the AD cohort vs. HC. Second, we performed a feature selection in a k-fold or leave-one-subject-out cross validation when training the classifier models. In both scenarios, significant features were selected using several successive statistical analysis methods. First, the normality distribution of each feature was examined using the Shapiro-Wilk test [31]. Then, t-test and Mann-Whitney U test were used for comparison of features between the HC and AD groups for features with normal and non-normal distributions, respectively [32], [33]. A significant difference was considered for *p*-value *<* 0.05. Finally, we selected a set of optimal features by performing a correlation analysis [33]. For this purpose, we identified any two features with a correlation coefficient of *>* 90% and selected the feature with the lowest *p*-value.

#### 6) Machine Learning Model:

We used a support vector machine (SVM) to discriminate AD vs. HC subjects based on the comprehensive TUG features. SVM is a supervised binary classifier that separates data with a linear separation [34]. When the data are not linearly separable, a kernel function is used to map the data into a new high-dimensional space where the mapped data are separated linearly [35]. Depending on the kernel type, SVM has several hyper-parameters that need to be adjusted. In the present study, we explored using a linear and Gaussian radial basis function (RBF) kernel with gamma (γ) parameter. Another parameter that needs adjusting is the regularization parameter (C), which allows SVM to tradeoff between misclassification and overfitting. We used a grid-search method to determine the optimal SVM kernel function and hyper-parameters $\gamma \in 2^{\left\{-4,\ldots ,4\right\}}$ and $C\in 2^{\left\{-4,\ldots ,4\right\}}$. For our analysis purposes, we used five-fold and leave-one-subject out cross validation for selecting the kernel and hyper-parameters. The classifiers’ performance was evaluated using several quantitative metrics of accuracy, sensitivity, precision, specificity, and F-score [36].

## III. Results

We applied our developed method on the skeletal data of 38 AD and 47 HC subjects who successfully performed the TUG test in front of the Kinect V.2 camera. In total, 61 features were extracted from each subject’s skeletal data. These features were adjusted for age and GDS score confounders. Table II shows the mean and standard deviation of the extracted features for the HC and AD groups and the *p*-values before and after adjusting for age and GDS score. Out of the 61 extracted features, 41 of them were significantly different between the two groups, which was decreased to 32 after adjusting for age and GDS score. When this process was repeated in a five-fold cross validation, these numbers decreased to 32 and 26 for before and after adjusting, respectively. Table III and Table IV show the number of extracted, significant, and selected features from different subtasks using the entire dataset and the training set of the five-fold cross validation for before and after the adjustment, respectively. The features that became insignificant after adjusting for age and GDS score were the duration of sit-to-stand subtask, mean and median of swing time, median of the double support time, mean and median of step width, mean and median of step time symmetry, and variability of stride time regularity.

Table II indicates that the duration of the TUG test and its subtasks were significantly greater for AD subjects except for the duration of the sit-to-stand subtask, which became insignificant after adjusting for age and GDS score although it remained greater in the AD cohort than in the HC cohort. The velocity of the stand-to-sit, walking, and turning subtasks were lower in AD subjects. Similarly, there were several significant gait features indicating a lower functional performance among the AD subjects in comparison to the performances of the HC group. For example, AD subjects spent more time in single and double stance phases and the step and stride times were greater than HC subjects. As shown in Table IV, two features from stand-to-sit, and 8 features from the walking, and two features from turning subtasks were selected for discriminating HC and AD subjects after adjusting for age and GDS and based on training data in the five-fold cross-validation.

**Fig. 9.**
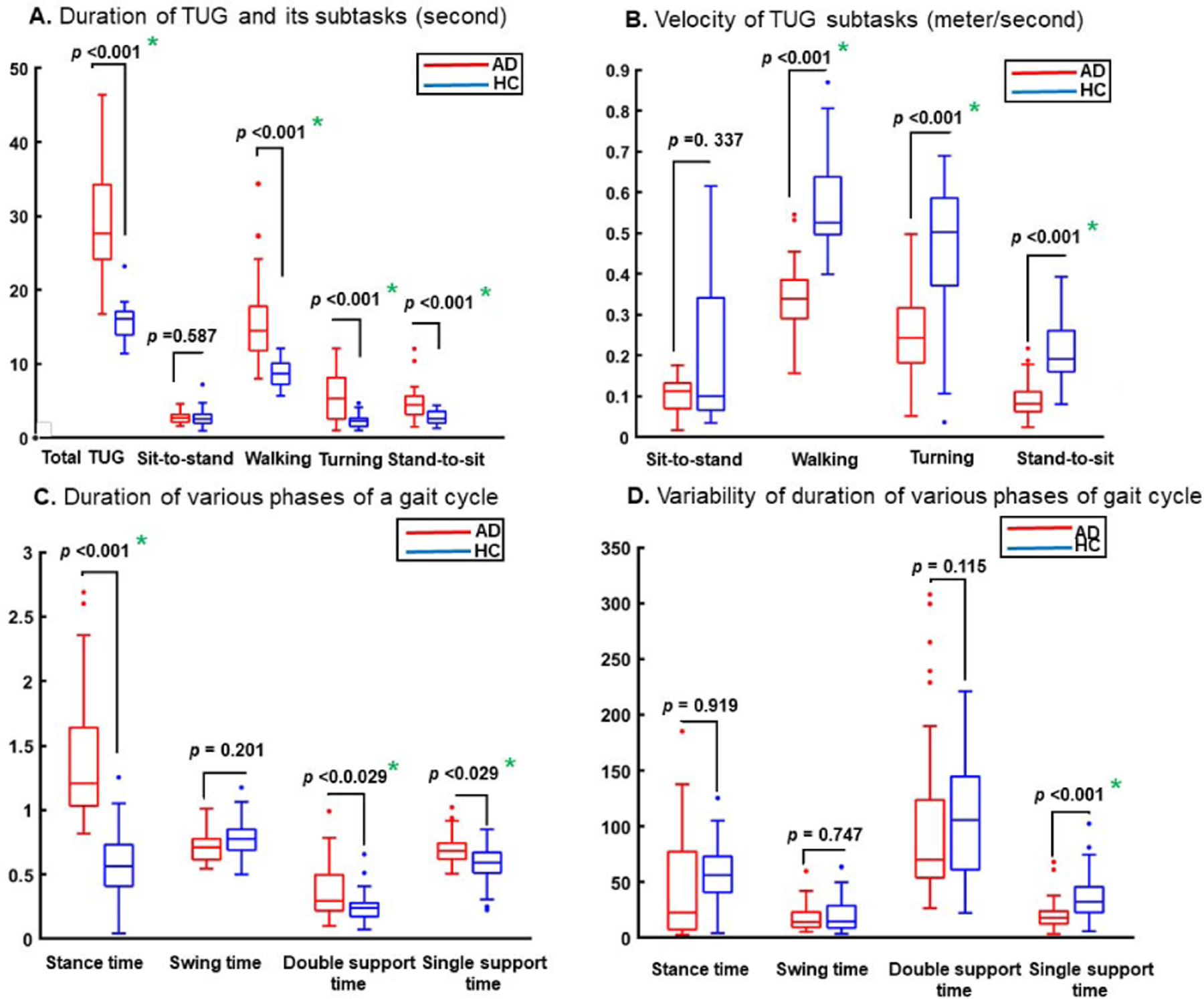
Comparison of different features from the TUG subtasks for HC vs. AD after adjusting for age and GDS score. The outliers are shown with circles and the significant difference is presented by an asterisk. The *p*-values are provided for each comparison.

**Fig. 10.**
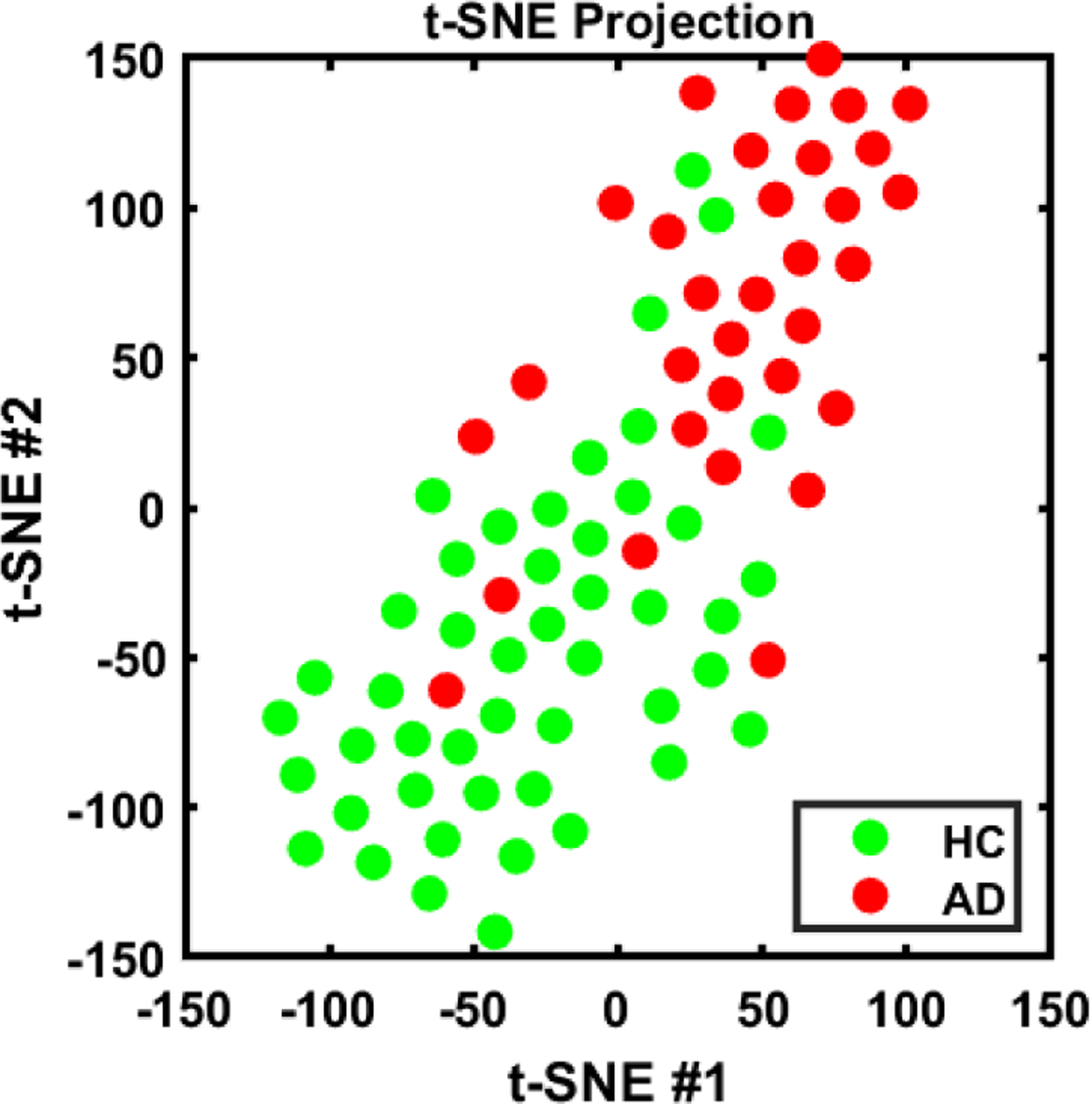
t-distributed stochastic neighbor embedding (t-SNE) of extracted data for AD and HC participants.

Fig. 9 shows the boxplots of the duration of the TUG subtasks and phases of gait cycle for HC vs. AD. Panel A provides the duration of each subtask of sit-to-stand, walking, turning, and stand-to-sit; Panel B the velocity of these subtasks; Panel C the timing of different phases of gait cycles, which are stance, swing, single-support, and dual-support. and Panel D provides the variability of the duration of these four gait phases. We also plotted the feature representation in 2-D using t-SNE (t-distributed stochastic neighbor embedding) method [37]. Fig.10 shows the t-SNE projection of extracted features to a 2-D space. As can been, the extracted features from the TUG test are generally different between AD and HC groups. The majority of the AD and HC subjects were separated from each other using the selected features although a few of the subjects are close to the boarder.

Five-fold and leave-one-subject out SVM classifiers using a linear and RBF kernel were trained and tested as explained in Section 2. Table V shows the average and standard deviation of accuracy and other evaluation metrics of five-fold cross-validation for detecting AD and HC. The results show that SVM with the RBF kernel has a better performance for detecting the AD subjects vs. HC from their TUG test with the accuracy of 97.75% and F-score of 97.67%. Table V also provides the performance of the leave-one-subject out cross-validation SVM, which has a higher accuracy of 98.68% and F-score of 98.67%. A precision of 100% indicates that all the healthy control participants were correctly detected. It is worth mentioning that if we did not adjust for age and GDS score, precision was indeed less than 100% indicating that the model may confuse low performance due to age or the other confounding factors with AD.

**Fig. 11.**
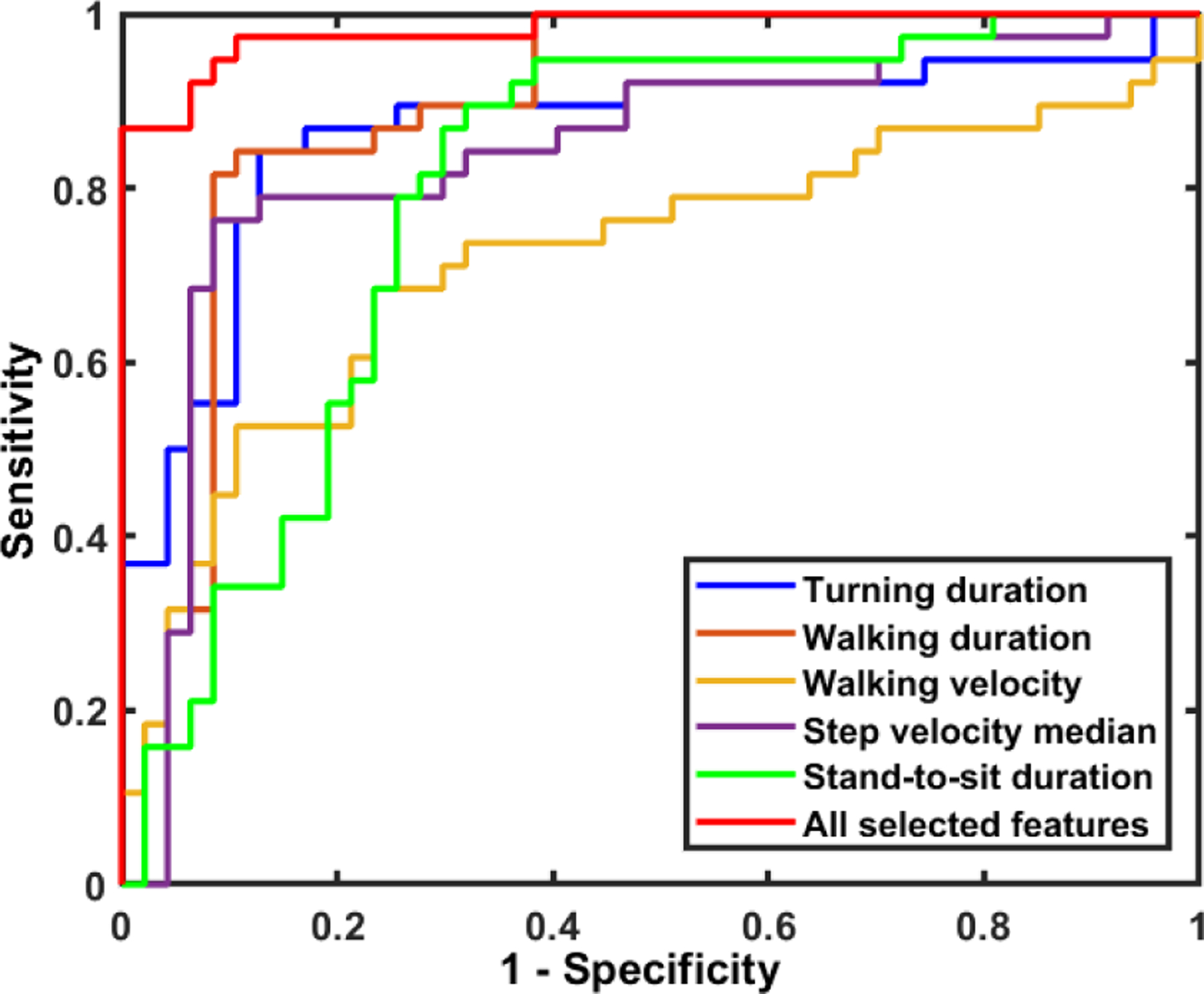
The ROC curves for detecting AD vs. HC using the top five significant features separately and all the selected features integrated via SVM.

Fig.11 shows Receiver Operating Characteristic Curve (ROC) for the top five significant features (i.e., lowest *p*-value) separately and the SVM classifier with leave-one-subject out cross-validation. As expected, the machine learning approach improved the discrimination power between AD vs. HC by integrating the significant features.

## IV. Discussion

Widespread evaluation of at-risk people for AD is a pressing challenge due to the complexity and cost of the assessment methods. We developed a method based on technology and the TUG test to address this challenge by making such evaluations without a significant complexity. We collected skeletal data from 38 AD and 47 HC subjects while performing TUG in front of a Kinect V.2 camera. Signal processing and descriptive statistical analysis were used to extract a set of significant features from different subtasks of TUG. We used machine learning to determine AD vs. HC subjects objectively.

Our main observation was that comprehensive analyses of TUG provided several significant features beyond just using the total duration of TUG using a stopwatch (Table II). First, we found that the overall time of TUG was significantly higher in the AD group than in the HC individuals, which was consistent with previous studies [7], [10], [38]. However, the total TUG time was not selected as the optimal feature since it was highly correlated with walking time, but the latter had a lower *p*-value and more discriminative value. Moreover, our analysis indicated that the duration of each TUG was significantly higher in AD vs. HC except for the sit-to-stand subtask (Fig. 9 Panel A). Additionally, the duration of the TUG test and walking subtasks were correlated but the latter provided a higher discrimination for AD vs. HC. Our analysis of gait subtask confirmed the results of previous studies from straight walking tests [4], [39]–[41] that AD individuals had significantly smaller step lengths and less average velocity in comparison to HC (Fig. 9 Panel B). We also observed that mean, median, and variability of gait characteristics were significantly different between the AD and HC participants indicating that several important features of the TUG test will be missed if only the duration of the TUG test is considered for assessing AD individuals (Table II and Fig. 9 Panel C and Panel D). Finally, among the velocity of the sit-to-stand, walking, and stand-to-sit subtasks, velocity of turning and the stand-to-sit subtask was selected mainly because other velocity parameters were significantly correlated with other features like time of walking but had a lower discrimination value (i.e., a higher *p*-value). Few studies have used sensing technologies to analyze the TUG test for cognitive impairment assessment. Mirelman *et al.* [12] used an inertial sensor with accelerometer and gyroscope and measured 20 features from 67 MCI and 280 HC individuals as they performed the TUG test. Their finding did not show a significant difference between the two groups except for a longer duration of turn-to-walk subtask for the MCI subjects [12]. However, they did not consider a comprehensive analysis of TUG, missing the analysis of subtasks’ velocity and features of step, stride and gait cycle. In another work, Wang *et al.* [13] used inertial sensors to extract 6 features from 21 AD and 25 HC individuals. They only extracted the overall time of TUG, time of sit-to-stand and stand-to-sit, as well as stride time, stance, and swing time during walking subtask. Their results agreed with our observations indicating that the performance of the AD group was weaker than the HC group. Ansai *et al.* [14] also examined TUG in detail using a system with seven cameras, which characterized body movement by tracking 15 markers mounted on the subjects’ bodies. They extracted 16 features from 40 HC, 40 MCI, and 38 AD subjects and showed that there were significant differences between AD and HC groups for all subtasks of TUG except for the time of the sit-to-stand subtask [14]. They did not examine the walking subtask comprehensively and only measured features of sit to stand, turning, and stand to sit as well as the overall time of TUG.

In the present study, the overall time of TUG, eight features including the duration and velocity of each subtask as well as 52 features from walking subtask were considered. In comparison, we extracted 61 features from various subtasks of the TUG test performed by 38 AD and 47 HC participants. The number of our extracted features was higher than the mentioned studies while the population of the HC and AD groups was also larger than some studies like Wang *et al.* [13] and Ansai *et al.* [14]. Considering the similar features in our study to the ones considered in these previous studies, we made a consistent observation in most parts except for the duration of the sit-to-stand subtask. Like our study, Ansai *et al.* [14] showed that the duration of sit-to-stand subtask was not significantly higher in the AD patients than in the HC subjects (see Panel A of Fig.9). This is while Wang *et al.* [13] found significantly different time of sit to stand when compared the AD to HC cohort, which could be due to several factors such as differences in the participant characteristics or recording tools. Wang *et al.* [13] also did not consider the cofounder factors of age and GDS, which could explain the difference between their observations and the ones reported by Ansai *et al.* [14] and our study. A detailed comparison between our study and the work of Wang *et al.* [13] and Ansai *et al.* [14] is provided in the Supplementary documents S3.

Our study was the first to confirm the applicability of using a Kinect V.2 camera for comprehensive assessments of the TUG test towards discriminating AD from healthy individuals. Other studies for TUG assessments were based on different sensing technologies including inertial sensors [12], [13] or RGB cameras and mounted markers on subjects’ bodies [14]. However, such methods are not entirely unobtrusive. They require to mount several inertial sensors on the body or in the case of using RGB cameras, they need to place several markers on the body for tracking body joints. They require time-consuming calibrations for every use and advanced processing methods to reduce sensitivity to the sensor or marker misplacement. There is a trend towards using regular cameras for skeleton detection and tracking [39]–[44]. Although the cost of a regular camera is less than a Kinect camera, we see several limitations of using them for the application in hand. First, the methods used to estimate joints’ locations from RBG images are based on pose estimation algorithms such OpenPose and VNect [39], [40], which use significant amount of labelled data to train the detection models. However, the labeling process was performed using healthy participants’ data, and it may not be done by experts with anatomical knowledge. Such inconsistencies and inaccuracies can increase the error in the training data and subsequently decrease the performance of these algorithms in real situation [45], especially in older adults with neurodegenerative diseases [46]. Second, the joint detection and tracking models require high computing power in comparison to the methods used for extracting joints using depth camera like Kinect V.2 [42]. Another concern is the privacy of participants as these cameras record videos for extracting joints during offline or online processing which may not be possible in many research and clinical settings. Our study used only one Kinect V.2 camera to collect comprehensive information of the skeletal data from 25 joints of a person’s body as well as depth and RGB data.

Our study was one of the first to show that machine learning can provide an objective metric for the presence or absence of AD from the TUG assessment. The SVM classifier was developed based on the 12 significant features selected from different TUG subtasks with an average accuracy of 97.75% and F-score of 97.67% in five-fold cross-validation after adjusting for age and GDS. Also, the average accuracy and F-score were 98.68% and 98.67%, respectively, for leave-one-subject out cross validation. This is while all the prior sensor-based TUG assessment methods [12]–[14] have only investigated differences of various subtasks between their study groups using descriptive statistical analysis without providing objective discrimination using machine learning.

### Clinical applicability:

Our work has the potential to provide an objective cognitive impairment assessment based on performing a simple TUG test in front of a Kinect V.2 camera. It can be used as a pre-screening method to identify at-risk individuals and refer them to specialists as needed. Moreover, it can allow for widespread assessments in nursing homes or even remote at-home assessments.

### Study limitations and future work:

To remove recordings with excessive outliers that were not filtered out using the preprocessing step, we used visual inspections. In the future, we will use automated techniques to estimate the affected recordings, however, in this study, we did not want those estimations affect the quality of the analyses. We used clinically meaningful engineered features to assess gait and balance. Our future work will take advantages of advanced deep learning models such as convolutional neural network (CNN) to investigate the presence of additional features beyond our extracted features. In addition, further analysis in a larger cohort with various forms of dementia and people with MCI is needed to validate the clinical diagnostic value of the proposed solution.

## V. Conclusion

This paper presented a novel method addressing the need for a widespread and easy-to-use technology-based assessment of AD individuals. Skeletal data from 25 joints of 47 HC and 38 AD subjects were collected as they performed the TUG test in front of a Kinect V.2 camera. A series of data processing and features extraction algorithms were performed to extract 61 features from different TUG subtasks. Our feature selection method resulted in 12 significant features between AD and HC groups after adjusting the extracted features for age and GDS score. Finally, a machine learning classifier based on SVM was developed to detect AD from HC. The results showed that analyzing the changes of spatiotemporal information of the body joints recorded with a Kinect V.2 camera produced significant features from various subtasks of the TUG test. The average accuracy and F-score of the SVM classifier using five-fold cross validation were 97.75% and 97.67%, respectively, and 98.68% and 98.67% when assessed using leave-one-subject out cross validation. These findings confirmed that the comprehensive analysis of TUG using a Kinect V.2 camera and machine learning has the potential to be used as an easy and inexpensive complementary method for the detection and regular quantitative assessment of AD in clinical or home settings. Our future work extends this method to a larger cohort which includes individuals with MCI and other related disorders.

## Supplementary Material

Suplemental tables

## Figures and Tables

**TABLE I:** Demographic and Some Clinical Information of the Study Participants

Variables	HC (n=47)	AD (n=38)	p-value
Age, mean ± sd (year)	68.47 ± 2.60	75.75 ± 7.17	<0.001*
Female, n (%)	25 (53)	21 (55)	0.849
Weight, mean ± sd (Kg)	66.82 ± 7.78	69.25 ± 13.35	0.540
Height, mean ± sd (Cm)	164.71 ± 6.84	169.25 ± 9.91	0.170
Education, mean ± sd (year)	13.41 ± 2.80	12.08 ± 4.60	0.450
MMSE, mean ± sd	28.64 ± 1.06	23.66 ± 3.09	0.001*
MoCA, mean ± sd	27.59 ± 1.87	19.50 ± 7.13	<0.001*
GDS, mean ± sd	1.24 ± 1.25	6.30 ± 5.19	0.001*

**sd** = Standard Deviation; **MMSE** = Mini-Mental State Examination (maximum score, 30); **MoCA** = Montreal Cognitive Assessment (maximum score, 30); **GDS** = Geriatric Depression Scale (maximum score, 15).

**TABLE II:** Comparison of the Extracted TUG Features for HC and AD Groups Before and After Adjusting for Age and GDS Using the Entire Dataset

Subtask	Features	Before adjusting for age and GDS		*p*-value	After adjusting age and GDS			*p*-value
		HC	AD		HC	AD		
Total TUG	Duration of TUG (s)	15.32 ± 2.15	27.17 ± 6.45	<0.001*	15.72 ± 0.30	28.86 ± 4.93	<0.001*	
Sit-to-Stand	Duration (s)	2.491 ± 1.28	2.91 ± 0.92	0.040*	2.71 ± 0.80	2.76 ± 0.68	0.587	
	Vertical Velocity (m/s)	0.17 ± 0.15	0.1 ± 0.04	0.250	0.2 ± 0.03	0.1 ± 0.09	0.337	
Walking	Duration (s)	8.69 ± 1.63	15.91 ± 5.52	<0.001*	8.69 ± 0.34	15.91 ± 3.56	<0.001*	
Turning	Duration (s)	2.29 ± 0.90	5.75 ± 3.4	<0.001*	2.29 ± 0.78	5.75 ± 1.53	<0.001*	
	Velocity (m/s)	0.47 ± 0.15	0.25 ± 0.09	<0.001*	0.47 ± 0.21	0.25 ± 0.22	<0.001*	
Stand-to-Sit	Duration (s)	2.57 ± 0.88	4.18 ± 2.22	<0.001*	2.77 ± 0.80	4.89 ± 0.85	<0.001*	
	Vertical Velocity (m/s)	0.20 ± 0.06	0.1 ± 0.04	<0.001*	0.21 ± 0.04	0.09 ± 0.03	<0.001*	
Gait	Velocity (m/s)	0.57 ± 0.11	0.34 ± 0.09	<0.001*	0.57 ± 0.08	0.34 ± 0.09	<0.001*	
	Stance time mean (s)	0.96 ± 0.44	1.38 ± 0.50	<0.001*	0.96 ± 0.43	1.38 ± 0.50	<0.001*	
	Stance time variability (%)	37 ± 36	39 ± 37	0.602	37 ± 32	39 ± 36	0.919	
	Stance time median (s)	0.85 ± 0.4	1.16 ± 0.25	<0.001*	0.85 ± 0.41	1.16 ± 0.24	<0.001*	
	Swing time mean (s)	0.78 ± 0.14	0.71 ± 0.11	0.010*	0.78 ± 0.14	0.71 ± 10.24	0.201	
	Swing time variability (%)	19 ± 15	17 ± 12	0.652	19 ± 15	17 ± 14	0.747	
	Swing time median (s)	0.78 ± 0.14	0.72 ± 0.11	0.012*	0.78 ± 0.14	0.72 ± 0.23	0.153	
	Double support time mean (s)	0.24 ± 0.10	0.36 ± 0.21	0.009*	0.24 ± 0.16	0.36 ± 0.13	0.029*	
	Double support time variability (%)	106 ± 46	100 ± 78	0.107	106 ± 0.46	100 ± 178	0.115	
	Double support time median (s)	0.15 ± 0.08	0.24 ± 0.12	<0.001*	0.15 ± 0.07	0.24 ± 0.17	0.143	
	Single support time mean (s)	0.58 ± 0.13	0.7 ± 0.12	<0.001*	0.58 ± 0.18	0.7 ± 0.26	0.029*	
	Single support time variability (%)	35 ± 20	20 ± 14	<0.001*	35 ± 21	20 ± 13	<0.001*	
	Single support time median (s)	0.59 ± 0.15	0.72 ± 0.12	<0.001*	0.59 ± 0.20	0.72 ± 0.23	0.011*	
	Step number	9.66 ± 1.92	14.42 ± 4.14	<0.001*	9.66 ± 0.19	14.42 ± 2.17	<0.001*	
	Step length mean (cm)	40.07 ± 5.78	31.17 ± 7.17	<0.001*	40.07 ± 3.92	31.17 ± 5.20	<0.001*	
	Step length variability (%)	29 ± 15	24 ± 9	0.127	29 ± 14	24 ± 17	0.148	
	Step length median (cm)	43.19 ± 5.92	32.59 ± 7.55	<0.001*	43.19 ± 4.08	32.59 ± 5.58	<0.001*	
	Step time mean (s)	0.85 ± 0.12	1 ± 0.16	<0.001*	0.85 ± 0.14	1 ± 0.16	<0.001*	
	Step time variability (%)	32 ± 14	34 ± 25	0.510	32 ± 15	34 ± 12	0.488	
	Step time median (s)	0.81 ± 0.09	0.96 ± 0.15	<0.001*	0.81 ± 0.14	0.96 ± 0.19	<0.001*	
	Step velocity mean (cm/s)	52.52 ± 9.98	35.19 ± 9.88	<0.001*	52.52 ± 8.05	35.19 ± 7.92	<0.001*	
	Step velocity variability (%)	43 ± 18	36 ± 14	0.038*	43 ± 18	36 ± 14	0.048*	
	Step velocity median (cm/s)	53.37 ± 9.91	34.35 ± 8.08	<0.001*	53.37 ± 8.01	34.35 ± 6.11	<0.001*	
	Step width mean (m)	0.13 ± 0.04	0.15 ± 0.03	0.004*	0.13 ± 0.08	0.15 ± 0.05	0.337	
	Step width variability (%)	26 ± 11	19 ± 13	<0.001*	26 ± 12	19 ± 15	0.008*	
	Step width median (m)	0.13 ± 0.04	0.15 ± 0.03	0.013*	0.13 ± 0.03	0.15 ± 0.04	0.333	
	Step height mean (m)	0.11 ± 0.04	0.1 ± 0.02	0.125	0.11 ± 0.07	0.1 ± 0.01	0.624	
	Step height variability (%)	34 ± 12	32 ± 8	0.781	34 ± 12	32 ± 8	0.926	
	Step height median (m)	0.11 ± 0.04	0.11 ± 0.03	0.241	0.11 ± 0.08	0.11 ± 0.02	0.533	
	Step frequency (step number/min)	65.54 ± 10	55.92 ± 9.12	<0.001*	65.54 ± 8.06	55.92 ± 7.18	<0.001*	
	Step length symmetry mean	0.74 ± 0.14	0.79 ± 0.07	0.201	0.74 ± 0.14	0.79 ± 0.20	0.159	
	Step length symmetry variability (%)	34 ± 26	20 ± 12	0.042*	34 ± 26	20 ± 11	0.036*	
	Step length symmetry median	0.77 ± 0.17	0.82 ± 0.07	0.891	0.77 ± 0.16	0.82 ± 0.19	0.143	
	Step time symmetry mean	0.68 ± 0.08	0.7 ± 0.10	<0.001*	0.68 ± 0.35	0.71 ± 0.10	0.477	
	Step time symmetry variability (%)	31 ± 14	28 ± 11	0.267	31 ± 18	28 ± 13	0.460	
	Step time symmetry median	0.72 ± 0.09	0.77 ± 0.11	<0.001*	0.72 ± 0.14	0.77 ± 0.11	0.279	
	Stride number	4.45 ± 0.83	6.92 ± 2.06	<0.001*	4.45 ± 0.82	6.92 ± 0.11	<0.001*	
	Stride time mean (s)	1.7 ± 0.25	2 ± 0.34	<0.001*	1.75 ± 0.25	2 ± 0.34	<0.001*	
	Stride time variability (%)	21 ± 12	23 ± 22	0.624	21 ± 13	23 ± 13	0.477	
	Stride time median (s)	1.65 ± 0.24	1.9 ± 0.33	<0.001*	1.65 ± 0.26	1.9 ± 0.34	<0.001*	
	Stride length mean (cm)	80.43 ± 10.95	62.79 ± 14.26	<0.001*	80.43 ± 9.06	62.79 ± 12.29	<0.001*	
	Stride length variability (%)	21 ± 12	18 ± 9	0.482	21 ± 13	18 ± 17	0.563	
	Stride length median (cm)	82.73 ± 13.01	64.54 ± 15.32	<0.001*	82.73 ± 11.1	64.54 ± 13.35	<0.001*	
	Stride velocity mean (cm/s)	49.98 ± 8.95	33.89 ± 9	<0.001*	49.98 ± 0.72	33.89 ± 7.03	<0.001*	
	Stride velocity variability (%)	28 ± 14	26 ± 11	0.498	28 ± 14	26 ± 12	0.477	
	Stride velocity median (cm/s)	50.77 ± 10.19	34.24 ± 8.46	<0.001*	50.77 ± 8.24	34.24 ± 6.49	<0.001*	
	Stride length regularity mean	0.77 ± 0.14	0.83 ± 0.09	0.105	0.77 ± 0.20	0.83 ± 0.13	0.153	
	Stride length regularity variability (%)	10 ± 9	9 ± 8	0.162	10 ± 3	9 ± 5	0.884	
	Stride length regularity median	0.78 ± 0.14	0.84 ± 0.09	0.069	0.78 ± 0.22	0.84 ± 0.13	0.113	
	Stride time regularity mean	0.78 ± 0.11	0.83 ± 0.08	0.069	0.78 ± 0.21	0.83 ± 0.21	0.351	
	Stride time regularity variability (%)	8 ± 3	13 ± 4	0.043*	8 ± 8	13 ± 4	0.398	
	Stride time regularity median	0.78 ± 0.11	0.85 ± 0.08	0.019	0.78 ± 0.24	0.85 ± 0.24	0.249	
	Stride frequency (stride number/min)	30.5 ± 4.62	26.82 ± 4.58	<0.001*	30.5 ± 2.67	26.82 ± 2.69	<0.001*	

Data are mean ± SD; The significant differences were considered forp-value < 0.05, and it is shown with * in the table

* The significant differences were considered forp-value < 0.05, and it is shown with in the table

**TABLE III:** Number of the Features Selected From Each TUG Subtask Based on the Entire Dataset

		TUG time	Sit-to-stand	Walking time	Turning	Stand-to-sit	Gait features				Total
							mean	med	CoV	others	
**Before adjusting age and GDS**	Extracted features	1	2	1	2	2	16	16	16	5	61
	Significant features	1	1	1	2	2	12	12	5	5	41
	Selected feature	1	0	1	2	2	5	5	5	2	23
**After adjusting age and GDS**	Extracted features	1	2	1	2	2	16	16	16	5	61
	Significant features	1	0	1	2	2	9	8	4	5	32
	Selected feature	0	0	1	2	2	3	6	4	2	20

**mean** = mean of features, **med** = median of feature, **CoV** = variability, **others** = number of steps, step frequency, number of strides, stride frequency, velocity

**TABLE IV:** Number of the Selected Features for Each TUG Subtask Based on the Five-Fold Cross Validation During Classifier Training

		TUG time	Sit-to-stand	Walking time	Turning	Stand-to-sit	Gait features				Total
							mean	med	CoV	others	
**Before adjusting age and GDS**	Extracted features	1	2	1	2	2	16	16	16	5	61
	Significant features	1	0	1	2	2	10	10	2	4	32
	Selected feature	1	0	1	2	2	5	3	2	1	17
**After adjusting age and GDS**	Extracted features	1	2	1	2	2	16	16	16	5	61
	Significant features	1	0	1	2	2	7	7	1	5	26
	Selected feature	0	0	1	2	2	1	4	1	1	12

**mean =** mean of features, **med =** median of feature, CoV = variability, **others =** number of steps, step frequency, number of strides, stride frequency, velocity

**TABLE V:** Classification Performance for Detecting AD Vs. HC

Cross-validation type	Kernel	Evaluation Metrics				
		Accuracy	Sensitivity	Precision	Specificity	F-score
**Five-fold**	Linear	97.5 ± 3.12	95 ± 6.25	100 ± 0	96.36 ± 4.55	97.14 ± 3.57
	RBF	97.75 ±1.56	96.5 ± 3.12	100 ± 0	97 ± 2.5	97.67 ± 1.67
**Leave-one-subject out**	Linear	93.67	89.47	97.14	92	93.15
	REF	98.68	97.37	100	97.92	98.67

Five-fold cross-validation: mean ± sd (%). Leave-one-subject out cross validation: mean (%)
