# Supplementary material for: Alzheimer’s Disease Detection Using Comprehensive Analysis of Timed Up and Go Test via Kinect V.2 Camera and Machine Learning
Source: IEEE Trans Neural Syst Rehabil Eng. Author manuscript; Available in PMC 2024 Jan 7. (PMC10771634; doi:10.1109/TNSRE.2022.3181252)
Supplement: Suplemental tables [file NIHMS1949036-supplement-Suplemental_tables.pdf]

Supplementary for “Alzheimer disease detection using comprehensive analysis of Timed Up and Go test via Kinect V.2 camera and machine learning”

S1. Noise and filtering

Various types of noises such as white noise, quantization noise, and spike noise can affect the quality of the collected data using digital devices like Kinect V.2 camera. In our work, we used a six-order Butterworth filter with 3 Hz cut-off frequency to remove such noise. Supplemental Fig.1 shows a sample signal before and after filtering. As can be seen, the noise of the right ankle joint in the y-direction has been removed using this filtering process.

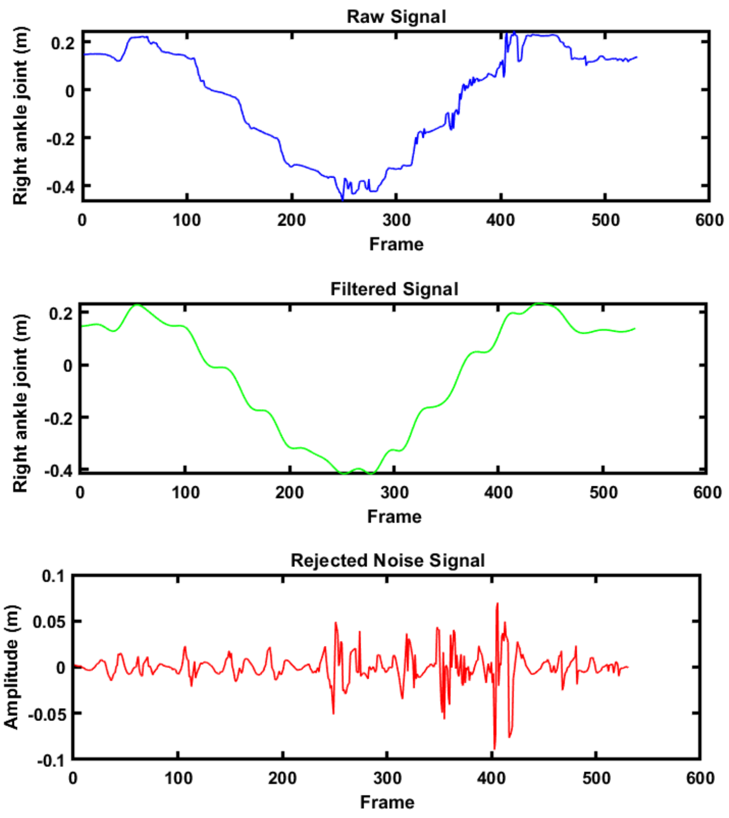

Supplemental Fig. 1. The raw recorded signal of a right ankle joint during the TUG test, the filtered signal, and the rejected noise using a six-order Butterworth filter with 3 Hz cut-off frequency.

S2. Visual inspections for detecting artifacts

We visually inspected the filtered signals to identify and delete the recordings which had a significant amount of noise that could not be removed using the filtering process. For this purpose, we inspected the ankle joint signal because it had two simple conditions during TUG tests: stance and swing phases. In the stance phase, the ankle joint position is constant, and during the swing phase, it changes gradually. Thus, we can easily notice the higher level of change in the stance and swing phases of the ankle joint in the presence of high noise. Supplemental Fig.2 shows three noisy samples of the right ankle joint signals. The pink rectangles indicated the artifacts in the form of sharp transitions that were not removed with the filtering process. These records were too noisy to extract accurate gait features and were removed from the analysis.

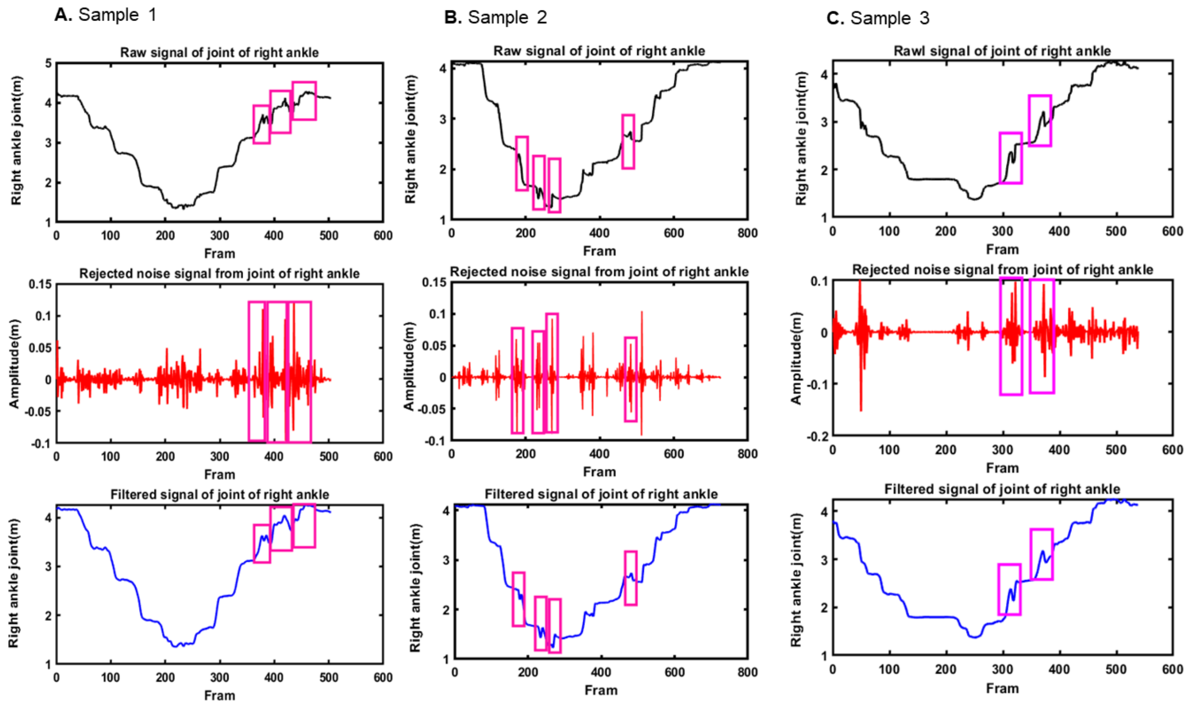

Supplemental Fig. 2. Three samples which were removed from the analysis because of the presence of the artifact in the filtered signals, as confirmed by visual inspection.

S3. Comparative analysis with other sensor-based AD assessment studies

Few studies have examined the TUG test comprehensively for the detection of AD (Alzheimer's disease). Wang *et al.* (2015) and Ansai *et al.* (2019) examined the TUG test and its subtask using sensor technology for AD detection. There are some similarities and differences between our study and those two studies. Here, we compared these studies' tools, methods, and results with our findings.

Recording tool: Ansai *et al.* (2019) focused on analyzing TUG and its subtask using the Qualisys system, which consists of seven cameras and 15 markers mounted on the subjects' bodies, and Wang *et al.* (2015) used three inertial-sensor-based wearable devices (made up of accelerometer, gyroscope, and remote controller) mounted on waist, right and left foot while we used skeletal data recorded with a single RGBD camera named Kinect V.2 camera.

Feature extraction: Ansai *et al*. (2019) extracted 46 features during the transition (sit-to-stand, turning, stand-to-sit, walking forward and back). Their features mainly were kinematic values like max and average of the trunk in different directions and velocity in different directions, especially for subtasks of turning and turn-to-sit which include transition. Wang *et al.* (2015) also extracted 6 features: TUG time, sit-to-stand, stand-to-sit, and stride, stance and swing phases during walking. In comparison, we extracted mostly clinical features, including 61 spatiotemporal features during the walking subtask as well as duration and velocity of other subtasks of TUG. Several extracted features like time of TUG and subtasks and the number of steps were common features between our study and studies done by Ansai *et al.* (2019).

Feature analysis: We also examined using machine learning to automatically discriminate HC (Healthy control) from AD subjects using selected features while Wang *et al.* (2015) and Ansai *et al*. (2019) did not.

Findings: The proposed method by Ansai *et al.* (2019) provided new valuable metrics using a more complex system. Wang *et al.* (2015) also proposed an automatic system for TUG analysis using wearable devices and showed that this system could be used for discriminating between AD and HC subjects. To make a clearer comparison between our study and some previous studies like Wang *et al.* (2015) and Ansai *et al.* (2019), we also created a table comparing the extracted features and findings. Supplemental Table 1 summarizes the extracted features from subtasks of TUG in our study and previous studies done by Wang *et al*. (2015) and Ansai *et al.* (2019) which examined the subtasks of TUG for discrimination between HC and AD older adults. Supplemental Table 1 shows the features extracted and findings in our study and those studies.

Supplemental Table 1. A comparative analysis of the existing studies for using TUG for AD detection.

Subtask	Features	Studies			Finding
		Wang *et al.* (2015)	Ansai *et al.* (2019)	Our study	
Total TUG	Duration of TUG (s)	✓	✓	✓	↑ for AD
Sit-to-Stand	Duration (s)	✓	✓	✓	↑ for AD [13] while no significant difference was reported by [14] and our study
	Vertical Velocity (m/s)	✗	✓	✓	No significant difference
	Range of motion	✗	✓	✗	No significant difference
Walking	Duration (s)	✗	✗	✓	↑ for AD
Turning	Duration (s)	✗	✓	✓	↑ for AD
	Velocity (m/s)	✗	✓	✓	↑ for AD
	Step number	✗	✓	✗	↑ for AD
Stand-to-Sit	Duration (s)	✓	✓	✓	↑ for AD
	Vertical Velocity (m/s)	✗	✓	✓	↑ for AD
	Range of motion	✗	✓	✗	No significant difference
	Step number	✗	✓	✗	↑ for AD
Gait	Velocity (m/s)	✗	✓	✓	↓ for AD
	Stance time mean (s)	✓	✗	✓	↑ for AD
	Stance time variability (%)	✗	✗	✓	No significant difference
	Stance time median (s)	✗	✗	✓	↑ for AD
	Maximum peak from heel strike until the maximum peak of the ankle during stance phase	✗	✓	✗	No significant difference
	Minimum peak from heel strike until the maximum peak of the ankle during stance phase	✗	✓	✗	No significant difference
	Average peak from heel strike until the maximum peak of the ankle during stance phase	✗	✓	✗	No significant difference
	Maximum peak from heel strike until the maximum peak of the knee during stance phase	✗	✓	✗	No significant difference
	Minimum peak from heel strike until the maximum peak of the knee during stance phase	✗	✓	✗	↑ for AD along the pitch axis
	Average peak from heel strike until the maximum peak of the knee during stance phase	✗	✓	✗	↑ for AD along the pitch axis
	Maximum peak from heel strike until the maximum peak of the hip during stance phase	✗	✓	✗	No significant difference
	Minimum peak from heel strike until the maximum peak of the hip during stance phase	✗	✓	✗	↓ for AD along the pitch axis
	Average peak from heel strike until the maximum peak of the hip during stance phase	✗	✓	✗	↓ for AD along the pitch axis
	Range of motion from heel strike until the maximum peak of the ankle during stance phase	✗	✓	✗	No significant difference

	Range of motion from heel strike until the maximum peak of the knee during stance phase	✗	✓	✗	No significant difference
	Range of motion from heel strike until the maximum peak of the hip during stance phase	✗	✓	✗	No significant difference
	Swing time mean (s)	✓	✗	✓	↑ for AD
	Swing time variability (%)	✗	✗	✓	No significant difference
	Swing time median (s)	✗	✗	✓	↑ for AD
	Double support time mean (s)	✗	✗	✓	↑ for AD
	Double support time variability (%)	✗	✗	✓	No significant difference
	Double support time median (s)	✗	✗	✓	↑ for AD
	Single support time mean (s)	✗	✗	✓	↑ for AD
	Single support time variability (%)	✗	✗	✓	↓ for AD
	Single support time median (s)	✗	✗	✓	↑ for AD
	Step number	✗	✓	✓	[14] found no significant difference while our study showed significant ↑for AD
	First step length	✗	✓	✗	No significant difference
	Step length mean (cm)	✗	✗	✓	↓ for AD
	Step length variability (%)	✗	✗	✓	No significant difference
	Step length median (cm)	✗	✗	✓	↓ for AD
	First step time	✗	✓	✗	No significant difference
	Step time mean (s)	✗	✗	✓	↑ for AD
	Step time variability (%)	✗	✗	✓	No significant difference
	Step time median (s)	✗	✗	✓	↑ for AD
	Step velocity mean (cm/s)	✗	✗	✓	↓ for AD
	Step velocity variability (%)	✗	✗	✓	↓ for AD
	Step velocity median (cm/s)	✗	✗	✓	↓ for AD
	Step width mean (m)	✗	✗	✓	↑ for AD
	Step width variability (%)	✗	✗	✓	↓ for AD
	Step width median (m)	✗	✗	✓	↑ for AD
	Step height mean (m)	✗	✗	✓	No significant difference
	Step height variability (%)	✗	✗	✓	No significant difference
	Step height median (m)	✗	✗	✓	No significant difference
	Step frequency (step number/min)	✗	✗	✓	↓ for AD
	Step length symmetry mean	✗	✗	✓	No significant difference
	Step length symmetry variability (%)	✗	✗	✓	↓ for AD
	Step length symmetry median	✗	✗	✓	No significant difference
	Step time symmetry mean	✗	✗	✓	↑ for AD
	Step time symmetry variability (%)	✗	✗	✓	No significant difference
	Step time symmetry median	✗	✗	✓	↑ for AD
	Stride number	✗	✗	✓	↓ for AD
	Stride time mean (s)	✓	✗	✓	↑ for AD
	Stride time variability (%)	✗	✗	✓	No significant difference
	Stride time median (s)	✗	✗	✓	↑ for AD
	Stride length mean (cm)	✗	✗	✓	↓ for AD
	Stride length variability (%)	✗	✗	✓	No significant difference
	Stride length median (cm)	✗	✗	✓	↓ for AD
	Stride velocity mean (cm/s)	✗	✗	✓	↓ for AD
	Stride velocity variability (%)	✗	✗	✓	No significant difference
	Stride velocity median (cm/s)	✗	✗	✓	↓ for AD
	Stride length regularity mean	✗	✗	✓	No significant difference
	Stride length regularity variability (%)	✗	✗	✓	No significant difference
	Stride length regularity median	✗	✗	✓	No significant difference

	Stride time regularity mean	✗	✗	✓	No significant difference
	Stride time regularity variability (%)	✗	✗	✓	↑ for AD
	Stride time regularity median	✗	✗	✓	No significant difference
	Stride frequency (stride number/min)	✗	✗	✓	↓ for AD

✓ = extracted; ✗ = non extracted; ↑ = significant increase among AD subjects in comparison to HC subjects;
↓ = significant decrease among AD subjects in comparison to HC subjects.

References

- Wang, W. H., Chung, P. C., Yang, G. L., Lin, C. W., Hsu, Y. L., Pai, M. C. An inertial sensor based balance and gait analysis system. 2015 IEEE International Symposium on Circuits and Systems (ISCAS), Lisbon, Portugal, 24-27 May 2015, pp. 2636-2639.
- Ansai, J. H., Andrade, L. P., Rossi, P. G., Nakagawa, T. H., Vale, F. A., Rebelatto, J. R. Differences in timed up and go subtasks between older people with mild cognitive impairment and mild Alzheimer's disease. Motor Control 2019, 23, 1-12.
